# A New Index of Energy Dissipation Considering Time Factor under the Impact Loads

**DOI:** 10.3390/ma15041443

**Published:** 2022-02-15

**Authors:** Xuesong Wang, Lianjun Guo, Zhenyang Xu, Junxiang Wang, Ding Deng, Jinglong Xu, Zhihang Hu

**Affiliations:** 1 School of Architecture and Civil Engineering, Shenyang University of Technology, Shenyang 110870, China; wangxs@smail.sut.edu.cn (X.W.); w.j.x.good@163.com (J.W.); dingdeng@smail.sut.edu.cn (D.D.); xjl1306575661@foxmail.com (J.X.); 2 College of Mining Engineering, University of Science and Technology Liaoning, Anshan 114051, China; xuzhenyang10@foxmail.com (Z.X.); huzhihang123@foxmail.com (Z.H.); 3 Chengyuan Mining Development Co., Ltd., Liaoyang 111299, China

**Keywords:** split Hopkinson pressure bar, dynamic mechanical properties, dynamic stress equilibrium, energy time density

## Abstract

Rock failure phenomena are accompanied by abundant energy variation, and the energy dissipation can explain the dynamic mechanical characteristics of the rock. In this study, a series of granite specimens (a total of 60) with different aspect ratios were dynamically loaded by a split Hopkinson pressure bar (SHPB) to explain the energy dissipation and the rock-crushing degree under dynamic load. A new index, namely energy time density (*w*_td_), is proposed to evaluate the energy dissipation considering the time factor. The relationships between strain rate, energy time density, and specific energy absorption are analyzed. A metric (*K*_u_) is defined to describe the degree of rock fragmentation quantitatively. The correlations of fractal dimension and *K*_u_ with different impact pressures are compared. It was concluded that there is a noticeable peak point in the energy time density curve. The energy time density of the stress equilibrium point is three times that of the peak point. The energy time density declines after the peak point, then the energy consumption density tends to be stable. The linear relationship between strain rate and peak point energy time density is stronger. The new index can describe energy dissipation well under dynamic loading. In addition, the experimental results indicate that the degree of crush *K*_u_ can describe the degree of crush, and the effect of fractal dimension to quantify the fracture characteristics of the rocks is less good in this test. The crushing degree of rocks increases with the increase of strain rate. Furthermore, the prediction effect of energy time density is better than that of strain rate about *K*_u_.

## 1. Introduction

Dynamic loads caused by blasting, drilling, earthquakes, etc., which act on and damage rocks lead to potential safety hazards in mining, water conservancy, underground space utilization, etc. [1,2,3,4,5]. It is essential to understand the effect of dynamic loading on the failure characterization and mechanism of rock materials for the safety of rock engineering. Split Hopkinson pressure bars (SHPB) have been used to study dynamic rock loading to investigate dynamic properties and energy dissipation of rocks [6,7,8,9,10]. Under the SHPB test, Yang et al. [11] investigated the dynamic mechanical behaviour of sandstone at different temperatures, and Li et al. [12] demonstrated that microwave irradiation has a significant effect on the dynamic tensile response of the rock. In addition, a study by Liu et al. [13] demonstrated the existence of a significant strain rate effect on the energy dissipation pattern of composite rocks under impact loading by applying the SHPB test to layered composite rock. Gong et al. [14] confirmed the linear energy dissipation law and considered the critical incident energy to be 0.29~0.33 MJ/m^3^ through dynamic fracture tests. During the SHPB test, there is a rich variation in the rock under dynamic loading, and the energy dissipation quantified by energy consumption density has attracted widespread attention. The critical parameters for calculating energy consumption density are incident energy, transmitted energy, and reflected energy. Zheng et al. [15] analyzed the relationship between the cyclic load threshold and energy evolution law of damaged sandstone. Zhou et al. [16] found two evident sub-regions for the energy partition by using the modified split Hopkinson pressure bar to discuss the fracturing process of rock under the axial static pre-stresses varying from 0 to 75 MPa. In the research of Li [17] et al., the energy calculation was used to explain the law of energy dissipation density under different strain rates for cemented backfill. Li et al. [18] found a correlation between the various patterns of energy dissipations and the development of microcracks. In the study of Zhang et al. [19], the energy dissipation density as an important parameter of the damage variable can better express the dynamic stress–strain relationship of sandstones under real-time high temperatures. In evaluating energy, energy consumption density as a standard metric has its disadvantage in describing the time effect. Energy time density [20] was used as a new metric to study the energy dissipation in dynamic loading to solve the problem. Liu [21] considered that the energy time density and incident energy show an upward trend. Following a further study by Pan [22], it was demonstrated that energy absorption reaches its highest level when the joint angle is 45°. Li [23] found that the penetration rate of a joint can lead to the increase of energy time density. The fractal dimension is the most common index to evaluate the crushing degree of rocks under dynamic loads. The study of Wang et al. [24] proved that the higher the fractal dimension, the higher the crushing energy density of rocks, which indicates the crushing degree of rock more completely. Zhao et al. [25] developed the continuum statistical constitutive model and described the fragmentation characteristics by fractal theory. Although the fractal dimension is the essential evaluation parameter, the effectiveness of this evaluation is unsatisfactory. This is mainly because the generalization of the regularity of this approach is controversial, and the fractal dimension exhibits an insignificant linear relationship with the staining rate [24,25]. In addition, quadratic functions have been used to fit the curve between strain rate and fractal dimension [26]. In summary, energy consumption density and fractal dimension are the parameters used to evaluate the degree of fragmentation in the study of energy dissipation under dynamic loading. The above approaches have had many achievements, however, gaps still exist. For example, (1) the assessment of energy dissipation is imperfect in terms of time effects and (2) the fractal dimension cannot accurately describe the degree of rock fragmentation.

Based on the above, we propose a new index (energy-time density *w*_td_) for evaluating the energy dissipation of rock in the stress equilibrium phase under the impact load. The relationship of strain rate, stress difference, energy time density, specific energy absorption, and stress equilibrium factor were analyzed for different impact air pressure. The new metric *K*_u_ can be used to quantify the fragmentation of rocks. The results show that the new index can describe energy dissipation more accurately and *K*_u_ can clearly define the degree of fragmentation. The article can provide a new analysis method for SHPB tests and provide some research references.

## 2. Materials and Methods

### 2.1. Split Hopkinson Pressure Bars (SHPB) Test System

**Figure 1:**
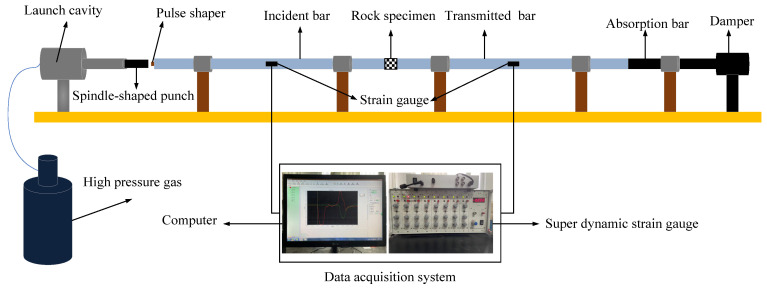
Diagram of the split Hopkinson pressure bars (SHPB) experimental system.

Hopkinson developed the SHPB loading system in 1914. The rock specimens were placed between the incident bar and transmitted bar then the punch was launched by the high-pressure gas in the test. On the premise of one-dimensional stress wave theory, the stress–strain of specimens can be calculated by the measured strain of the incident bar and transmitter bar. In this study, all laboratory tests were carried out in the SHPB loading system in the University of Science and Technology Liaoning, as shown in Figure 1. The incident bar was 2100 mm, the transmitted bar was 1800 mm, the absorbing bar was 800 mm; the bars were also made of high-strength steel with a diameter of 50 mm, and the elastic modulus was 210 GPa. The air pressure range was 0.12 MPa, 0.15 MPa, 0.18 MPa, 0.24 MPa in the test.

### 2.2. Specimen Preparation

**Figure 2:**
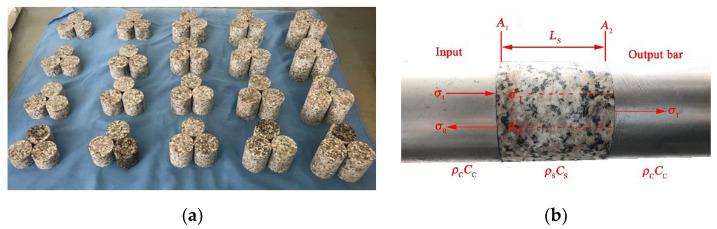
Specimens of granite and experiment system: (**a**) the granite specimens, (**b**) the granite between the incident bar and transmitted bar.

Granite specimens were obtained from Mount.Taihang mining area in Xinyang, Henan Province, China. The all-granite specimens were from the same homogeneous rock. According to the standard of the International Society for Rock Mechanics, the specimen ends’ tolerances of evenness and non-parallelism were less than 0.02 mm. The specimens were manufactured into cylinders with a 50 mm diameter, the reflection coefficient was −0.55~0.52, and the aspect ratio of specimens was 0.6, 0.8, 1.0, 1.2, 1.4, as shown in Figure 2. The specimen ID was organized in the form of “N-A,” wherein “N” represents the aspect ratio of the specimen and “A” represents the air pressure. The mechanical parameters of the granite are listed in Table 1. In the test, three samples were tested under each condition and the average results were recorded.

### 2.3. Preparation of SHPB System before Formal Tests

**Figure 3:**
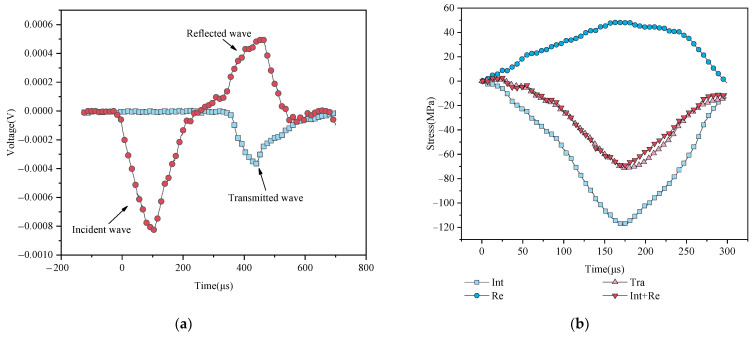
Trial experiment and dynamic stress equilibrium: (**a**) typical stress wave pattern of a tested rock specimen; (**b**) dynamic stress equilibrium.

Before formal tests, a typical test was performed to check the stress equilibrium. Figure 3a shows the typical stress wave pattern of a tested rock specimen, and the incident wave, transmitted wave, and reflected wave can meet the test requirements. Figure 3b shows the typical stress equilibrium test curve, meaning the requirements of stress equilibrium during dynamic loading.

### 2.4. Calculation of the Strain and Stress

In the SHPB test, the three-wave method [27,28] was used to analyze the dynamic characteristics of granite specimens.

The axial stress $\sigma_{s}$, strain $\varepsilon_{s}$ and strain rate ${\dot{\varepsilon }}_{s}$ can be calculated as Equation (1):

(1) $$\left\{ \begin{array}{l} \sigma_{s}=\frac{A_{0}E_{0}}{2A_{s}}\left(\varepsilon_{I}+\varepsilon_{R}+\varepsilon_{T}\right)\\ \varepsilon_{s}=\frac{C_{0}}{l_{s}}\int_{0}^{t}\left(\varepsilon_{I}-\varepsilon_{R}-\varepsilon_{T}\right)dt\\ {\dot{\varepsilon }}_{s}=\frac{C_{0}}{l_{s}}\left(\varepsilon_{I}-\varepsilon_{R}-\varepsilon_{T}\right) \end{array}\right.$$

where *A*_0_ and *E*_0_ are the cross-sectional areas and elastic modulus of the bar. *A*_s_, *C*_0_ and *A*_s_ are the cross-sectional area, P-wave velocity, and length of the specimen. $\varepsilon_{I}$, $\varepsilon_{R}$ and $\varepsilon_{T}$ are the incident, reflected, and transmitted strain of the test, respectively. $l_{s}$ is the length of the granite specimen.

In the SHPB test, the air pressure makes the bullet move, and the kinetic energy of the bullet is converted into the incident energy. Ignoring the kinetic energy of rock fragments and the heat exchange between the rock and outside, the energy consists of three parts: the first part of the energy return incident bar is carried by a reflected wave, the second arrives at the transmitted bar by a transmitted wave, and the last is absorbed by the granite specimen. The strain energy in bars [29] can be calculated as Equation (2):

(2) $$\{\begin{array}{c} W_{i}=\frac{A_{0}C_{0}}{E_{0}}\int \sigma_{i}^{2}dt=A_{0}E_{0}C_{0}\int \varepsilon_{i}^{2}dt\\ W_{r}=\frac{AC_{0}}{E_{0}}\int \sigma_{r}^{2}dt=A_{0}E_{0}C_{b}\int \varepsilon_{r}^{2}dt\\ W_{t}=\frac{A_{0}C_{0}}{E_{0}}\int \sigma_{t}^{2}dt=A_{0}E_{0}C_{0}\int \varepsilon_{t}^{2}dt \end{array}$$

where, $W_{i}$, $W_{r}$ and $W_{t}$ are then carried by the incident, reflected, and transmitted waves, respectively.

The calculation method of the absorbed energy of specimen $W_{d}$ [30] is shown in Equation (3):

(3) $$W_{d}=W_{i}-W_{r}-W_{t}$$

The index of energy consumption density *w*_s_ is introduced to evaluate the absorption of energy in rock, and the calculation is shown in Equation (4):

(4) $$\omega_{s}=\frac{W_{d}}{V}=\frac{A_{0}E_{0}C_{0}}{A_{s}l_{s}}\int [\varepsilon_{i}{\left(t\right)}^{2}-\varepsilon_{r}{\left(t\right)}^{2}-\varepsilon_{t}{\left(t\right)}^{2}]dt$$

where V is the volume of the granite specimen.

In the SHPB, the incident energy affects the dynamic characteristics of the specimen, and the energy magnitude has received widespread attention in previous studies. However, the action time of the energy also affects the dynamic characteristics of the specimen. This energy structure with time is determined as energy time density *w*_td_, and the calculation is shown in Equation (5):

(5) $$w_{\mathrm{td}}=\frac{w_{s}}{T_{w}}$$

where $T_{w}$ is the duration of the stress wave.

The functions of energy time density are shown in Equation (6):

(6) $$w_{\mathrm{td}}(t)=\frac{w_{s}(t)}{t}$$

## 3. Results and Analysis

In the SHPB test, the difference of wave impedance is between the elastic bar and the granite specimen. The transmission and reflection stress waves are complex in granite specimens and take up a certain time in the granite specimen. The time consumption $\tau_{s}$ can be calculated by the length of the specimen and the P-wave velocity $C_{s}$ as Equation (7) shows:

(7) $$\tau_{s}=\frac{L_{s}}{C_{s}}$$

This phenomenon in which the stress at both ends is different in dynamic loading determines rock specimens’ stress variation and equilibrium effects. The first method is stress difference $\alpha_{k}$ [2]. The stress difference $\alpha_{k}$ at both ends of the granite specimen is shown in Equation (8):

(8) $$\alpha_{k}=\frac{\Delta \sigma_{k}}{\bar{\sigma_{k}}}\times 100\%=\frac{T_{BS}\left[\sigma_{i}(t_{k})-\sigma_{i}(t_{k-1})\right]-F_{SB}\Delta \sigma_{k-1}}{T_{BS}\frac{\sigma_{i}(t_{k})+\sigma_{i}(t_{k-1})}{2}+F_{SB}{\bar{\sigma }}_{k-1}}\times 100\%$$

where $\Delta \sigma_{k}$ and $\bar{\sigma_{k}}$ are the stress difference and average value of stress at both ends of the rock specimen, respectively. $T_{BS}$ and $F_{SB}$ are the coefficient of transmission and reflection, respectively. $t_{k}$ and $\sigma_{i}(t_{k})$ are the *k*-th time and stress on the stress wave propagates in the granite specimen, respectively.

Another method is the stress equilibrium factor $\sigma_{\mathrm{eq}}$ [31] which can be calculated by Equation (9):

(9) $$\sigma_{\mathrm{eq}}=\frac{\sigma_{t}}{\sigma_{i}+\sigma_{r}}$$

When the stress equilibrium factor $\sigma_{\mathrm{eq}}$ is close to 1, the granite specimen has reached stress equilibrium. 

**Figure 4:**
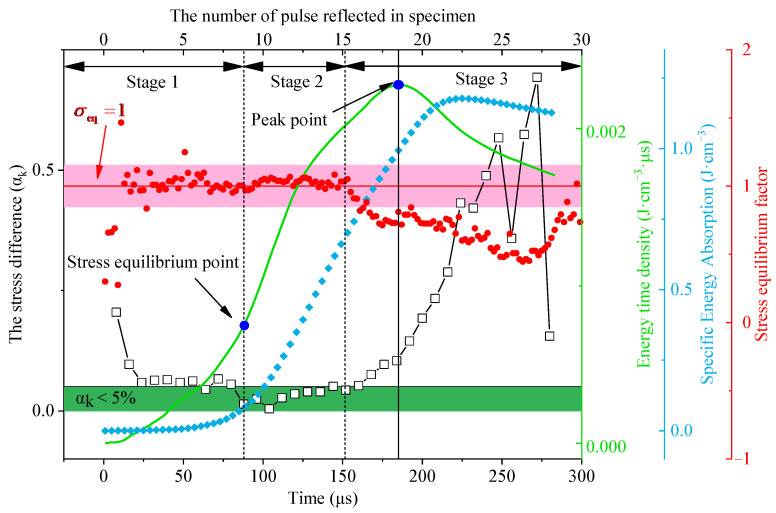
A typical dynamic test for stress equilibrium.

Figure 4 shows the typical test results that obtain the stress difference, energy time density, and specific energy absorption curve. The length of the granite specimen was 40 mm in the test and the P-wave velocity was 5000 m/s. Furthermore, the $\tau_{s}$ was 8 μs. The granite specimen reached the stress equilibrium when the stress difference $\alpha_{k}$ was less than 0.05 [2].

The stress difference curve in Figure 4 can be divided into three stages: stress superposition stage, stress equilibrium stage, and stress deterioration. In the stress superposition stage, the stress wave propagates repeatedly and is superimposed on the granite specimen. The stress difference at both ends of the specimen is large. The stress difference $\alpha_{k}$ is 0.20 at 8 μs (n = 1), and the distribution of stress is uneven in the granite rock. The incident energy causes stress concentration at the incident face of the rock in the stress superposition stage, and the microcrack and deformation occur in the locality of the rock at this moment. With the increasing of loading time, the stress difference gradually decreases, the first stress equilibrium is at 64 μs (n = 8), and the value oscillates in the next few microseconds. Then, the stress equilibrium stage begins and lasts for some time. The $\alpha_{k}$ is less than 0.05 from 88 μs to 162 μs, and the stress of both ends of the granite specimen is almost equal on this stage. The state of the rock meets the requirements of stress equilibrium in dynamic loading. Finally comes the stage of rock deterioration, and a lot of microcracks are activated by the absorbed energy in the rock. The $\alpha_{k}$ gradually increases in this stage. The variability of the $\sigma_{\mathrm{eq}}$ curve also proves the stress equilibrium under dynamic loading.

Because of the stress equilibrium is sensitive to time, the effect of a new index about the time factor under dynamic load should be discussed. In the specific energy absorption curve as shown in Figure 4, the variation trend shows a correlation with stress equilibrium. The specific energy absorption curve rises slowly in the stress superposition stage. In the stress equilibrium stage, the *w*_s_ rises significantly, and most of the absorbed energy in the rock is mainly absorbed in this stage. 

In the final stage, the variation of energy almost stops. The absorbed energy has reached saturation in the rock, and the curve of energy consumption density makes it difficult to express the time factor in dynamic loading intuitively. However, the effect of the time factor on energy absorption is shown by the energy time density curve. When the time is 64 μs, the rock reaches the first time stress equilibrium, and the energy time density is 7.48 × 10^−4^ J·cm^−3^·μs^−1^. A peak point appears in the curve. The energy time is 2.28 × 10^−3^ J·cm^−3^·μs^−1^ at 185 μs. The energy time density of the stress equilibrium point is three times that of peak point. The assimilation of energy mainly happens in the stress equilibrium stage. As Figure 4 shows, there is a peak point in the curve of energy time density and energy consumption density. The peak points appear at 188 μs and 224 μs, respectively. The curve of energy time density declines after the peak point; the energy consumption density tends to be stable in this stage. Therefore, the energy time density considers the time factor under the dynamic loading and can evaluate the energy absorption in the rock better than the energy consumption density.

### 3.1. Energy Analysis

**Figure 5:**
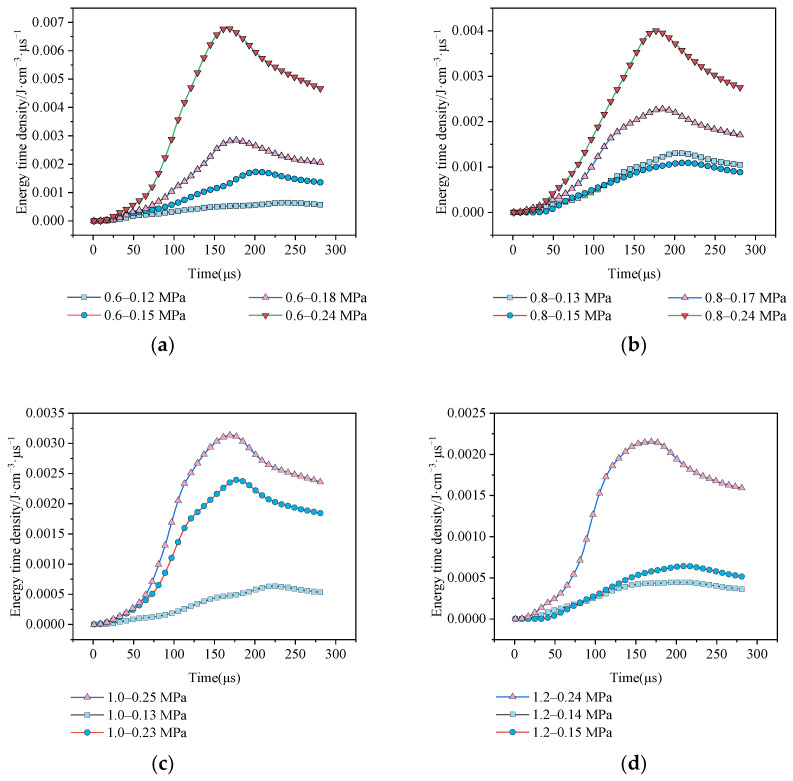

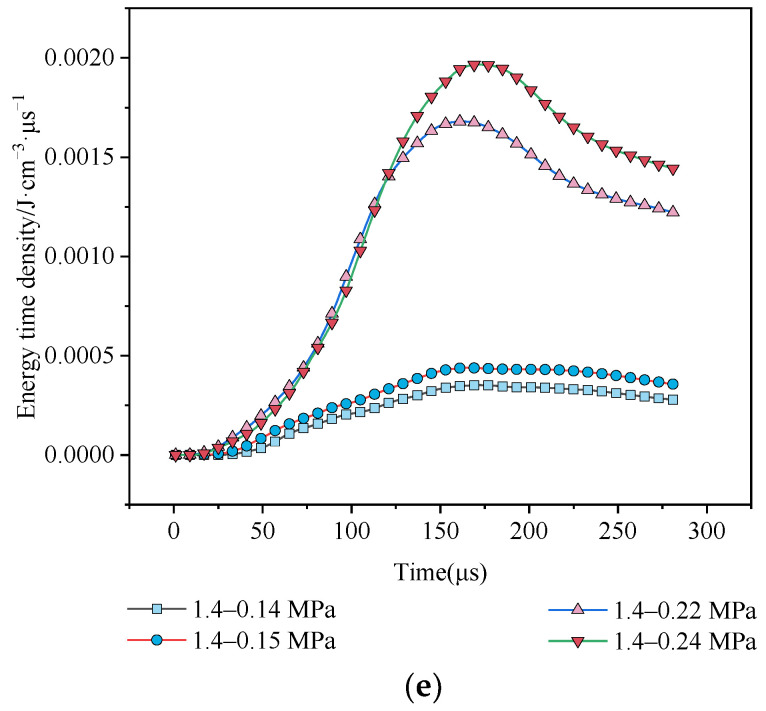
The time-energy time density curves: (**a**) aspect ratio is 0.6; (**b**) aspect ratio is 0.8; (**c**) aspect ratio is 1.0; (**d**) aspect ratio is 1.2; (**e**) aspect ratio is 1.4.

As Figure 5 shows, the trend of each group of curves are similar and the energy time density is stable for evaluating the energy absorption in the rock. The wtd is increased and then decreased and there are peak points in every curve. The peak points appear at around 150 μs. In the same group, the air pressure significantly affects the peak energy time density. The energy dissipation results are shown in Table 2.

**Figure 6:**
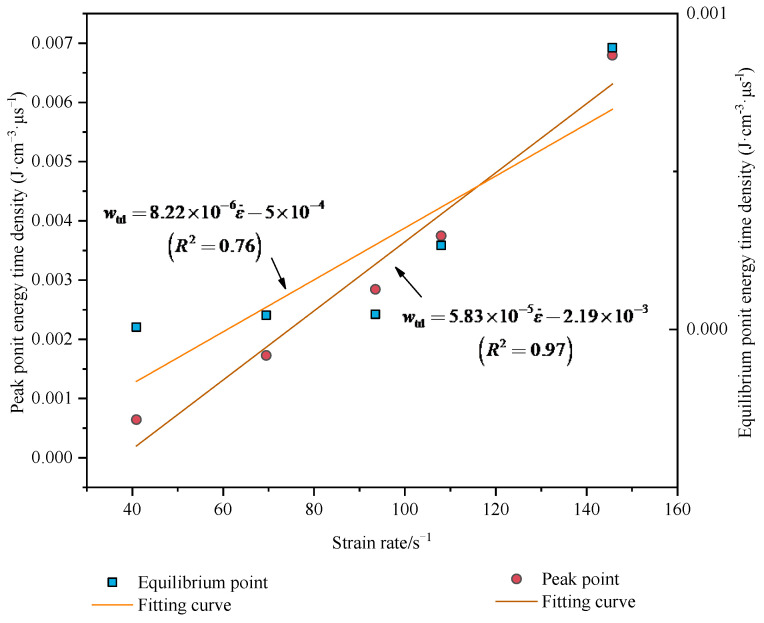
The curve of the equilibrium point and the peak point energy time density with strain rate.

The equilibrium point and peak point curves show the sensitivity of *w*_td_ to strain rate. As Figure 6 shows, there is a linear relationship between the equilibrium point energy time density and peak point energy time density with strain rate. The energy time density increases as the strain rate increases. The fitting equation is as follows:

(10) $$\begin{array}{cc} \begin{array}{l} E_{\mathrm{td}}=5.83\times 10^{-5}\dot{\varepsilon }-2.19\times 10^{-3}\left(R^{2}=0.97\right),\\ E_{\mathrm{td}}=8.22\times 10^{-6}\dot{\varepsilon }-5.01\times 10^{-4}\left(R^{2}=0.76\right) \end{array}\} & \end{array}$$

With the correlation coefficients *R*^2^ of 0.97 and 0.76 for the peak point energy time density and the equilibrium point energy time density, respectively, there was general regularity which is not very high for the equilibrium point energy time density. The reason is that stress distribution is uneven, and then the energy absorption is unstable before stress equilibrium. This is similar to the discussion for Figure 4. A more significant linear relationship for the peak point is that the *R*^2^ is 0.97. Therefore, the effect by which the peak point energy time density *w*_td_ evaluates the energy absorption of rock under dynamic loading is better.

**Figure 7:**
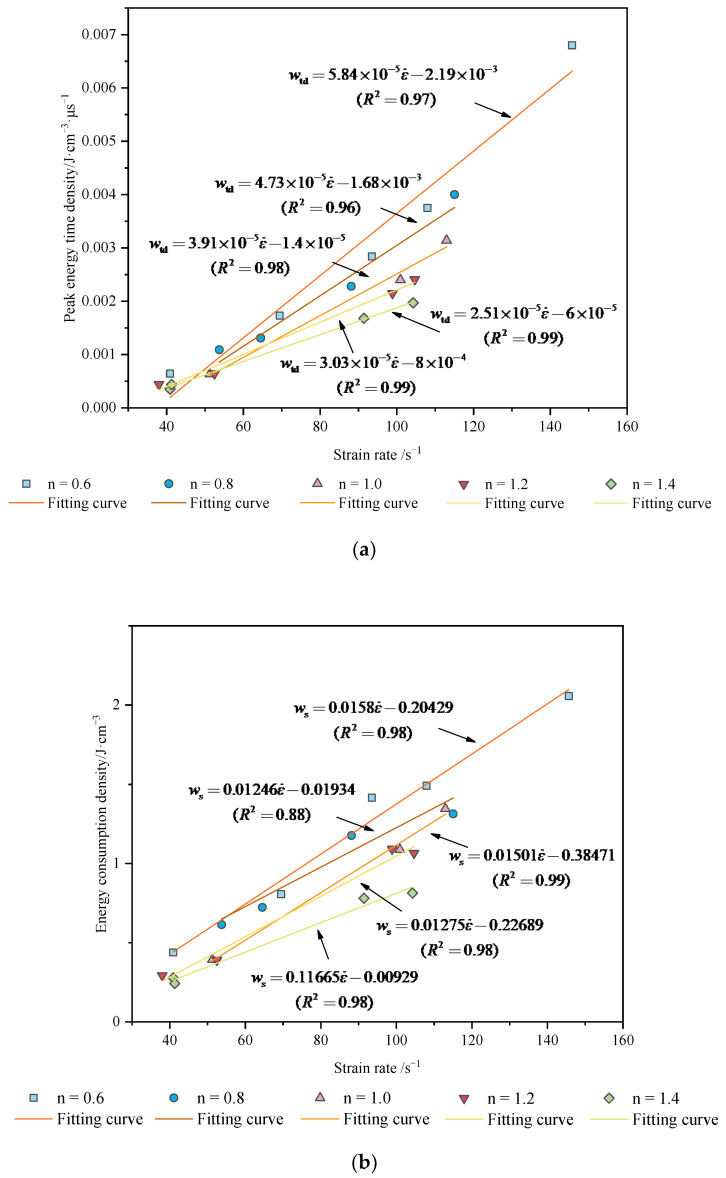
Plots of the relationship between the strain rate and energy absorption: (**a**) the peak energy time density curve; (**b**) the energy consumption density.

As seen from Figure 7, the strain rate has an apparent linear relationship with the peak energy time density and the energy consumption density of the different aspect ratios for granite specimens. The *w*_td_ of different aspect ratios are shown in the following equation:

(11) $$\begin{array}{c} \begin{array}{c} w_{\mathrm{td}-0.6}=5.84\times 10^{-5}\dot{\varepsilon }-2.19\times 10^{-3}\;(R^{2}=0.97),\\ w_{\mathrm{td}-0.8}=4.73\times 10^{-5}\dot{\varepsilon }-1.68\times 10^{-3}\;(R^{2}=0.96), \end{array}\\ w_{\mathrm{td}-1.0}=3.91\times 10^{-5}\dot{\varepsilon }-1.41\times 10^{-5}\;(R^{2}=0.98),\\ w_{\mathrm{td}-1.2}=3.03\times 10^{-5}\dot{\varepsilon }-8.13\times 10^{-4}\;(R^{2}=0.99),\\ w_{\mathrm{td}-1.4}=2.51\times 10^{-5}\dot{\varepsilon }-6.36\times 10^{-5}\;(R^{2}=0.99) \end{array}\}$$

The *w*_s_ of different aspect ratios are shown in the following equation:

(12) $$\begin{array}{c} \begin{array}{c} \begin{array}{c} w_{s-0.6}=0.01581\dot{\varepsilon }-0.20429\;(R^{2}=0.98),\\ w_{s-0.8}=0.01246\dot{\varepsilon }-0.01934\;(R^{2}=0.88), \end{array}\\ w_{s-1.0}=0.01501\dot{\varepsilon }-0.38471\;(R^{2}=0.99),\\ w_{s-1.2}=0.01275\dot{\varepsilon }-0.22689\;(R^{2}=0.98), \end{array}\\ w_{s-1.4}=0.11665\dot{\varepsilon }-0.00929\;(R^{2}=0.98) \end{array}\}$$

As Figure 7 shows, the two indexes can evaluate the energy abortion in rock, but the effect has a difference. The correlation coefficients *R*^2^ of 0.97, 0.96, 0.98, 0.99 and 0.99 for the energy time density and the *R*^2^ of energy consumption density are 0.98, 0.88, 0.99, 0.98 and 0.98. The results show that the fitting effect of energy time density is better, and prove the importance of the time factor in valuing energy dissipation, and so the new index can connect the loaded time and energy dissipation.

### 3.2. The Degree of Crush K_u_

The study of Rittinger [32] interpreted the energy absorption in rock as the increase of fracture surface, and the energy needed to generate new surface $E_{b}$ can be calculated by Equation (13):

(13) $$E_{b}=2\gamma S_{\Delta }$$

where $S_{\Delta }$ is the added surface area after rock fracture, and γ is the surface energy of rock.

Ideally, the crushing scale approaches 0, when the crushing of rock is complete. Every rock element can be considered the same cube or sphere of size *d*. 

$S_{0}$ is the initial area of rock, *V* is the volume of the rock, *d* is the target particle size at the time that the rock is crushed completely.

(14) $$S_{\Delta }=\frac{6V}{d}-S_{0}$$

Then, the energy of the rock crushed completely $U_{w}$ can be calculated by Equation (15):

(15) $$U_{w}=2\gamma (\frac{6V}{d}-S_{0})$$

In the SHPB test, it is difficult to crush the rock completely. The results are always incomplete, with rock fragmentation in the dynamic loading test. When incomplete, crushed rocks are broken to the target particle size. The crushing energy $U_{e}$ can be calculated by Equation (16):

(16) $$U_{e}=\gamma S_{\Delta }^{m}$$

where, $S_{\Delta }^{m}$ is the increase of surface. 

The surface area of all rock fragments $S_{m}$ can be calculated by the gradation curve:

(17) $$S_{m}=6V\int_{0}^{\infty }\frac{f(x)}{x}dx$$

where, f(x) is the function of a gradation curve. The proportion of rock volume is $p_{k}$ in the range of $x_{1}^{k}$ to $x_{2}^{k}$.

(18) $$p_{k}=\int_{x_{1}^{k}}^{x_{2}^{k}}f(x)dx$$

The average probability density function $\bar{f}(x)$ is:

(19) $$\bar{f}(x)=\frac{p_{k}}{x_{2}^{k}-x_{1}^{k}}$$

The surface area of rock fragments in the test results is:

(20) $$S_{\Delta }^{m}=\frac{6V}{d}-\int_{0}^{\infty }f(x)dx=6V(\frac{1}{d}-\sum_{k=1}^{M}\frac{p_{k}}{x_{2}^{k}-x_{1}^{k}}\ln\frac{x_{2}^{k}}{x_{1}^{k}})$$

When the remaining rocks are broken to the target particle size, the change of surface area $S_{\Delta }^{m}$ is:

(21) $$S_{\Delta }^{m}=\frac{6V}{d}-\int_{0}^{\infty }f(x)dx=6V(\frac{1}{d}-\sum_{k=1}^{M}\frac{p_{k}}{x_{2}^{k}-x_{1}^{k}}\ln\frac{x_{2}^{k}}{x_{1}^{k}})$$

The crushing energy of the remaining rocks is $U_{e}$:

(22) $$U_{e}=\gamma S_{\Delta }^{m}=\gamma 6V(\frac{1}{d}-\sum_{k=1}^{M}\frac{p_{k}}{x_{2}^{k}-x_{1}^{k}}\ln\frac{x_{2}^{k}}{x_{1}^{k}})$$

Therefore, the index of rock crushing about the target particle size $K_{u}$ can be defined:

(23) $$K_{u}=1-\frac{U_{e}}{U_{w}}$$

where *K*_u_ is the index of rock crushing characterized by the fracture condition of the rock under crushing scale *d*. In the unloaded rock, the function of gradation is $S_{0}$, and the $U_{e}=U_{w}$ and the *K*_u_ is 0 shows that the rock is intact under this condition. There is an ideal situation in which the rock consists of fragments with radius *d*, and $U_{e}$ is 0, which means that the rock is completely broken. When the polynomial of gradation is less than 1/d in Equation (22), the *K*_u_ is more than 1, which means the rock is excessively broken.

**Figure 8:**
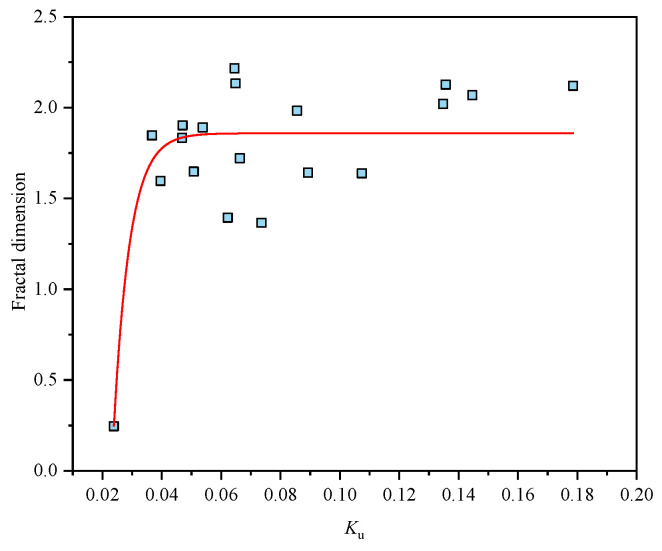
The curve of *K*_u_ and fractal dimension.

In previous studies [24], the results showed that the rock was more broken with the *D* increasing. As the *K*_u_-*D* curve shows in Figure 8, the *D* tends to increase with the *K*_u_ increasing, showing that the degree of crushing *K*_u_ can describe the degree of crushing in dynamic loading. The calculation method of fractal dimension is the same as in [24].

**Figure 9:**
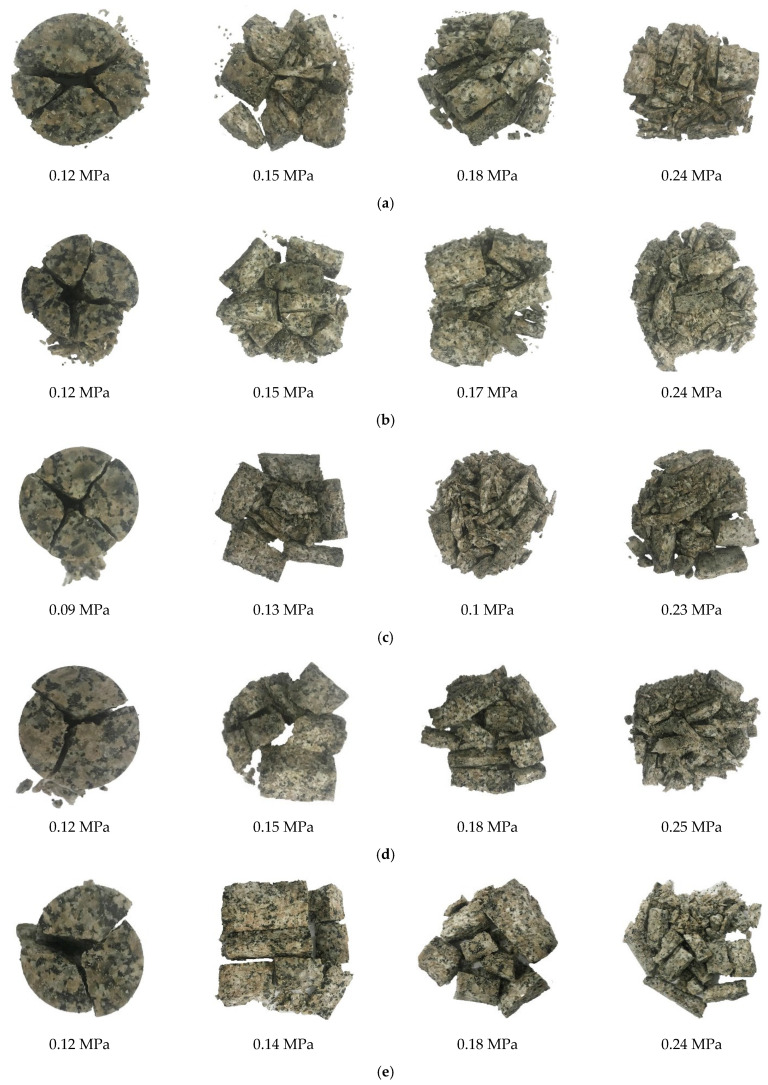
Damage pattern of granite specimen at different air pressures: (**a**) aspect ratio is 0.6; (**b**) aspect ratio is 0.8; (**c**) aspect ratio is 1.0; (**d**) aspect ratio is 1.2; (**e**) aspect ratio is 1.4.

**Figure 10:**
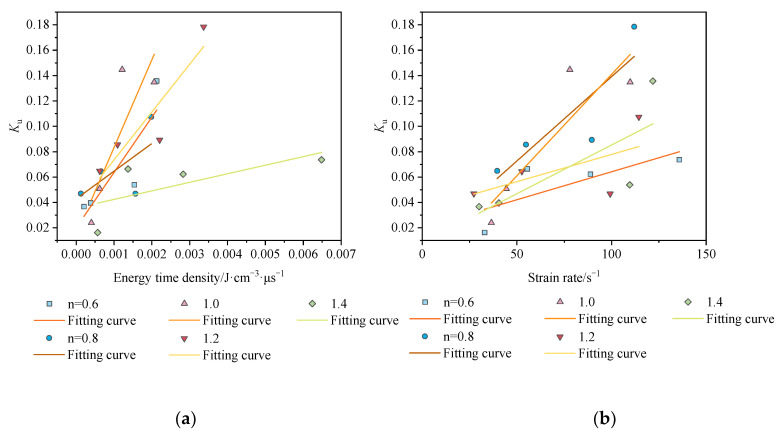

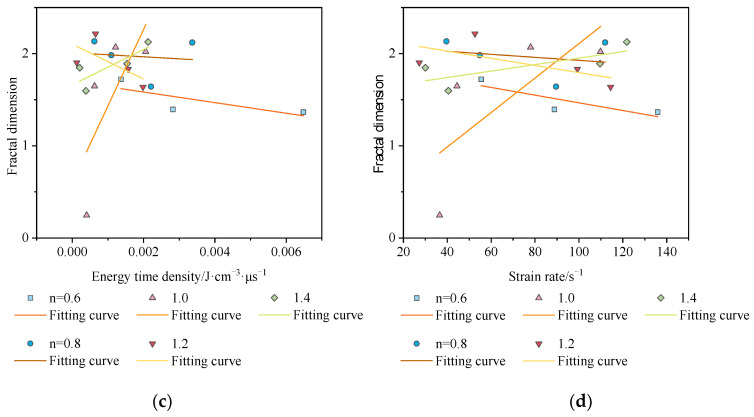
Plots of the relationship between the strain rate and degree of fragmentation: (**a**) the peak energy time density—*K*_u_ curve; (**b**) the strain rate—*K*_u_ curve; (**c**) the energy time density—*D* (**d**) the strain rate—*D*.

The fractal dimension *D*, the sieving results, and the *K*_u_ are calculated in Table 3, and Figure 9 shows that fragmentation becomes finer as air pressure increases. Therefore, the fragmentation is finer with the strain rate higher. When the air pressure is 0.12 MPa, the fragment of the granite specimen is generally large. It is finer under the 0.24 MPa air pressure. In Table 3, it is difficult to describe the degree of crushing by the sieve sizes. For example, the proportions of >26.5 mm sieve size are 78.21% and 76.94% in the Jobs “1.2–0.12” and “1.2–0.15”, respectively. The *K*_u_ can quantify the degree of crushing. To prove the stability of the *K*_u,_ the curves are shown in Figure 10.

As Figure 10 shows for previous studies [24,25] the crushing degree increases with the strain rate increasing. However, the fractal dimension is not highly correlated with strain and energy time density in this research. The trends are negatively correlated in Figure 10c,d, when the aspect ratio is 0.6, 0.8, and 1.2, respectively. The trends are a positive correlation in the remaining two cases. The correlation coefficients are 0.52 and 0.66. The results show that the effect of fractal dimensions to quantify the fracture characteristics of the rocks is poorer. It can be seen from Figure 10a,b, that the regularity of *K*_u_ with energy time density and strain rate is more pronounced. In Table 4, the correlation coefficients of strain rate–fractal dimension and energy time density–fractal dimension are 0.31~0.79 and 0.41~0.83. In most cases, the *K*_u_ can evaluate the degree of fracture characteristics well, and the energy time density achieves better prediction results than the strain rate.

## 4. Conclusions

In this paper, dynamic impact tests on granite with different aspect ratios were carried out under different energies. A new index of energy-time density was defined to evaluate the energy dissipation of rocks under dynamic loading. The degree of crush *K*_u_ was proposed to quantify the crushing of rocks in the test. This research provides a reference for further study of rock mechanics testing. The main conclusions are as follows:

(1) The changing trend of the new index (energy time density) and energy consumption density are similar; the peak point is more prominent and appears around 180 μs. The new index is more sensitive to energy dissipation.
(2) There are linear trends in the new index and energy consumption density with strain. The correlation coefficients *R*^2^ of energy time density and the *R*^2^ of energy consumption density are 0.96~0.99 and 0.88~0.99, and the correlation between the new index (energy time density) and strain rate is substantial.
(3) The fractal dimension is not highly correlated with strain and energy time density in this study. The degree of crush *K*_u_ can quantify fracture characteristics of the rock. The degree of crush *K*_u_ is 0.024 to 0.179 under the dynamic impact tests. The rock crushing degree’s evaluation effect is better than the fractal dimension.
(4) The *K*_u_ increases as the strain rate and the energy time density increase. In most cases, the energy time density achieved better prediction results than the strain rate. The correlation coefficients *R*^2^ are 0.31~0.79 and 0.41~0.83, respectively.

## Figures and Tables

**Table 1:** Mechanical parameters of granite under static load.

Density (kg/m^3^)	P-Wave Velocity (m/s)	Elastic Modulus (GPa)
2723	4888	36.68

**Table 2:** The energy dissipation results of the samples at different strain rates.

Specimen ID number	Incident Energy /J	Strain Rate /s^−1^	Dynamic Strength /MPa	Energy Time Density in the Peak Point /J·cm^−3^·μs^−l^	Energy Time Density in Equilibrium Point /J·cm^−3^·μs^−l^	Specific Energy Absorption /J·cm^−3^
0.6–0.12	186.34	33.44	85.91	0.0026	0.0008	0.50
0.6–0.15	236.27	59.54	109.34	0.0050	0.0009	0.71
0.6–0.18	282.42	84.38	103.18	0.0079	0.0019	0.95
0.6–0.24	332.76	132.01	122.72	0.0125	0.0066	1.57
0.8–0.13	242.57	56.22	106.68	0.0043	0.0017	0.74
0.8–0.15	235.79	51.25	85.66	0.0056	0.0026	0.30
0.8–0.24	281.73	104.26	121.76	0.0088	0.0024	0.88
1.0–0.13	227.76	43.80	113.26	0.0024	0.0008	0.42
1.0–0.23	317.43	89.77	139.48	0.0056	0.0034	1.23
1.0–0.25	343.08	101.36	131.26	0.0067	0.0047	1.51
1.2–0.14	229.02	30.55	114.39	0.0018	0.0014	0.40
1.2–0.15	229.89	49.60	87.49	0.0034	0.0010	0.30
1.2–0.24	341.45	86.04	149.26	0.0047	0.0035	1.43
1.4–0.14	221.63	38.00	102.78	0.0023	0.0010	0.25
1.4–0.15	213.66	35.10	97.69	0.0021	0.0018	0.36
1.4–0.22	325.35	78.59	143.27	0.0038	0.0031	1.17
1.4–0.24	328.18	93.91	127.41	0.0046	0.0034	1.09

**Table 3:** Table of impact test sieve sizes corresponding to the proportion.

Specimen Number	>26.5 mm	26.5 mm	19 mm	16 mm	13.2 mm	9.5 mm	4.75 mm	<2.36 mm	*D*	*K*_u_	Energy Time Density
0.6–0.12	74.37%	25.63%	-	-	-	-	-	-	-	0.016	0.0006
0.6–0.15	16.65%	54.81%	5.70%	7.15%	5.57%	2.28%	4.68%	3.16%	1.72	0.066	0.0014
0.6–0.18	-	42.72%	33.80%	12.03%	3.16%	2.03%	4.62%	1.65%	1.39	0.062	0.0028
0.6–0.24	19.24%	30.57%	4.05%	13.99%	16.27%	8.35%	6.20%	1.33%	1.37	0.074	0.0065
0.8–0.12	63.86%	23.27%	2.41%	1.80%	0.57%	0.24%	2.51%	5.35%	2.13	0.065	0.0006
0.8–0.15	20.15%	46.55%	14.76%	2.65%	3.22%	3.93%	1.94%	6.81%	1.98	0.086	0.0011
0.8–0.17	29.90%	10.50%	7.00%	16.65%	14.24%	14.62%	3.74%	3.36%	1.64	0.089	0.0022
0.8–0.24	-	14.71%	12.87%	13.62%	14.33%	20.86%	12.20%	11.40%	2.12	0.179	0.0034
1.0–0.09	90.57%	-	3.69%	3.61%	0.74%	1.12%	0.27%	-	0.25	0.024	0.0004
1.0–0.13	48.76%	32.66%	4.00%	4.07%	5.12%	1.59%	1.36%	2.44%	1.65	0.051	0.0006
1.0–0.18	-	24.40%	28.47%	11.48%	7.64%	8.53%	9.81%	9.66%	2.07	0.145	0.0012
1.0–0.23	-	37.63%	14.66%	4.58%	16.18%	10.90%	7.21%	8.84%	2.02	0.135	0.0021
1.2–0.12	78.21%	12.13%	3.82%	-	-	-	3.98%	1.86%	1.90	0.047	0.0001
1.2–0.15	76.94%	9.85%	5.94%	-	-	0.54%	1.01%	5.72%	2.22	0.065	0.0007
1.2–0.18	61.43%	29.88%	2.91%	-	-	2.65%	0.41%	2.72%	1.83	0.047	0.0016
1.2–0.25	-	26.91%	28.49%	6.89%	13.30%	11.56%	9.95%	2.91%	1.64	0.107	0.0020
1.4–0.12	89.72%	4.70%	-	0.94%	1.13%	1.42%	0.46%	1.64%	1.85	0.037	0.0002
1.4–0.14	66.45%	28.10%	-	-	-	2.98%	1.34%	1.13%	1.60	0.040	0.0004
1.4–0.18	49.97%	43.59%	-	-	-	-	4.11%	2.33%	1.89	0.054	0.0015
1.4–0.24	13.50%	33.41%	11.06%	9.98%	8.21%	6.66%	6.82%	10.36%	2.13	0.136	0.0021

**Table 4:** The correlation coefficients *R*^2^ of strain rate and energy time density with *K*_u_.

The *R*^2^ of Curve	Aspect Ratio				
	0.6	0.8	1.0	1.2	1.4
Strain rate—*K*_u_	0.58	0.75	0.79	0.37	0.60
Energy time density—*K*_u_	0.46	0.83	0.72	0.41	0.74
Strain rate—*D*	0.73	0.06	0.54	0.43	0.58
Energy time density—*D*	0.59	0.01	0.52	0.44	0.66
